# Improvement of Idiopathic Cardiomyopathy After Colon Clear

**DOI:** 10.14740/cr398e

**Published:** 2015-04-06

**Authors:** Randa M. Nasrat, Mohammad M. Nasrat, Abdullah M. Nasrat, Salwa A. Nasrat

**Affiliations:** a Department of Internal Medicine, Helwan Gen Hosp, Cairo, Egypt; b Department of Surgery, Balghsoon Clinics, Jeddah, KSA; c Department of Physiotherapy, Cardiac Surgery Academy, Cairo, Egypt

**Keywords:** Cardiomyopathy, *Helicobacter pylori*, Myocarditis, Senna purge

## Abstract

**Background:**

The study aimed to illustrate the effect of colon clear on idiopathic myocardial dysfunction. *Helicobacter pylori* colonized the stomach since an immemorial time, as if both the stomach and the bacterium used to live together in peace harmless to each other. *H. pylori* could migrate or get forced to migrate to the colon; antibiotics are seldom effective against extra-gastric *H. pylori* strains. The association of *H. pylori* and some cardiovascular diseases like myocarditis and cardiomyopaty has been sufficiently mentioned in literature. The role played by the increased mucosal production of inflammatory mediators (cytokines) induced by *H. pylori* among patients with ischemic heart diseases has been also clearly illustrated. The clinical association of gastritis and carditis is controversial. Active lymphocytic myocarditis manifested by intractable ventricular tachycardia, non-specific intra-ventricular block, and myocardial dysfunction has been described in a young woman infected with *H. pylori*; an immune influence has been emphasized in that patient as a possible etiology behind the development of autoimmune myocarditis. It has been reported also in literature that a possible role of autoimmunity induced by *H. pylori* in cardiomyopathy cannot be excluded.

**Methods:**

Three female patients with frank history of *H. pylori* dyspepsia and an age range of 41 - 47 years have developed myocaditis complicated with cardiomyopathy as confirmed by echocardiography (ECG) and magnetic resonance imaging (MRI). Existence of *H. pylori* in the colon was confirmed by *H. pylori* fecal antigen. Colon clear was done for them.

**Results:**

Symptomatic improvement and clinical recovery to sinus rhythm with minimal supra-ventricular extra-systoles occurred for all patients after colon clear. Patients continued improvement to normal cardiac tracing and normal left ventricular ejection function within further 3 - 4 weeks.

**Conclusion:**

Colon clear could be a simple and safe measure to improve changes in cardiac rhythm, heart rate and myocardial function developing in association with *H. pylori* due to inflammatory, toxic or immune reasons.

## Introduction

The widespread prevalence and the medical challenges constituted by *Helicobacter pylori*, namely its close relation to acid peptic disease, gastric carcinoma and lymphoma have led to the widely established medical concept that *H. pylori* eradication should be a necessary attempt [1, 2]. *H. pylori* colonized the stomach since an immemorial time [2], as if both the stomach and the bacterium used to live together in peace harmless to each other. *H. pylori* could migrate or get forced to migrate to the colon due to the influence of antibiotic violence [2-4]; antibiotics are seldom effective against extra-gastric *H. pylori* strains [5].

The latest reports in literature demonstrate a definite flare up of many medical challenges related to *H. pylori* through immune or different unknown reasons [6-9]. The association of *H. pylori* and some cardiovascular diseases like myocarditis and cardiomyopaty has been sufficiently mentioned in literature. The role played by the increased mucosal production of inflammatory mediators (cytokines) induced by *H. pylori* among patients with ischemic heart diseases has been also clearly illustrated [10-12].

The clinical association of gastritis and carditis is controversial; opinions are currently divided as whether it is the result of gastro-esophageal reflux or a proximal extension of *H. pylori* infection from the stomach [10]. Active lymphocytic myocarditis manifested by intractable ventricular tachycardia, non-specific intra-ventricular block, and myocardial dysfunction has been described in a young woman infected with *H. pylori*; an immune influence has been emphasized in that patient as a possible etiology behind the development of autoimmune myocarditis [11]. It has been also reported in literature that a possible role of autoimmunity induced by *H. pylori* in cardiomyopathy cannot be excluded [12]. Different reports in literature have confirmed the association of cytotoxin-associated gene A (cagA) positive *H. pylori* strains with many medical problems, and emphasized that cagA of *H. pylori* encodes a highly immunogenic and virulence-associated protein; the presence of this virulent gene in the body could affect the clinical outcome in many patients [2, 13].

The aim of this study was to illustrate the effect of colon clear on idiopathic myocardial dysfunction.

## Patients and Methods

### Design and setting

Multiple-case clinical study was done in Balghsoon Outpatient Clinics in Jeddah/Saudi Arabia during October 2012 - May 2013.

### Patients

Three female patients with frank long history of *H. pylori* dyspepsia and an age range of 41 -47 years have developed palpitation, chest tightness and breathing discomfort with frequent extra-systoles in the echocardiography (ECG). Shortly, two of them developed ventricular extra-systoles; an initial diagnosis of viral myocaditis complicated with idiopathic cardiomyopathy was made based upon ECG and magnetic resonance imaging (MRI). Their left ventricular ejedtion fraction was ranging between 29% and 30%. They were advised for insertion of an automated implantable cardioverter defibrillator (AICD) but they hesitated towards this procedure; therefore, they were put on medical treatment. Ventricular extra-systoles disappeared on medications but their distressing symptoms showed no improvement. *H. pylori* existence in the colon was confirmed by a reliable specific test, *H. pylori* fecal antigen [2]. Colon clear with the potent natural senna leaves purge was employed for them. Successful *H. pylori* eradication was confirmed by *H. pylori* fecal antigen test [2, 4]. *H. pylori* fecal antigen test was available from Acon Laboratory, USA, Batch No. HP8040008.

### Ethical considerations

An informed signed consent was taken from all patients, and they were free to quit the study whenever they like. All patients were allowed to follow their usual diet, medications and to lead their routine style of life. They were not requested to stop their medications but they did that gradually by their own because of physical uneasiness upon intake of pills. One of them stopped her medications all of a sudden as she missed them while traveling by road; she gave the expression of suddenly feeling health and freedom. The research proposal was approved and the study followed the rules of the Research Ethics Committee of King Faisal Specialized Hospital and Research Center in Jeddah, Saudi Arabia.

## Results

The three patients expressed immediate dramatic improvement after colon clear; they became free of any symptoms instantly after diarrhea was complete and they were able to exercise walking for continuous 1 h without fatigue or doing more than 400 m walk exercise in less than 6 min. One patient expressed “I am back myself again” while another patient said “now I can count my pulse”. Their ECG resumed a sinus rhythm with few supra-ventricular extra-systoles, around 7 - 9/min. The left ventricular ejection fraction became 47-49%. The three patients achieved complete recovery within 3 - 4 weeks; their left ventricular function improved to 53-55% and the ECG tracing became straight forward normal. Patients were not obliged to stop medications but later they did that by their own because of feeling uneasy with medications and they kept maintaining their condition stable even they were feeling better expressing further physical relief upon quit of medications. Patients were followed up for 12 months showing no recurrence as they were watching carefully their colonic condition.

The results of this study were compared with the records of seven female patients of rather similar age range (40 - 49 years) and rather similar physical and clinical parameters who have developed myocarditis and cardiomypathy confirmed by ECG and MRI, they were put on the following several medications: amiodarone hydrochloride 200 mg once daily, carvedilol 6.25 mg once daily and candesartan cilexetil 8 mg once daily together with two diuretic drugs (furosemide and spironolactone), a gstric sedative and aspirin in order to reduce symptoms and burden on the heart, while one patient was obliged to undergo AICD insertion because of risk on life. In spite of these measures, those patients were not getting much better as concerns palpitation, easy fatigability and chest discomfort.

The results of this study were further compared and confirmed also by the clinical study of one female patient aged 49.5 years who developed myocardititis and cardiomyopathy manifested with ventricular extra-systoles following a long history of *H. pylori* dyspepsia. She was advised for AICD insertion because of risk on her life but she was not able to afford the cost of this procedure, and she disappeared to reappear after 1 month symptom-less; she mentioned that she just followed camel milk intake with honey for 1 month. Her ECG was restored normal and her left ventricular ejection fraction improved from 31% to 49%.

**Figure 1:**
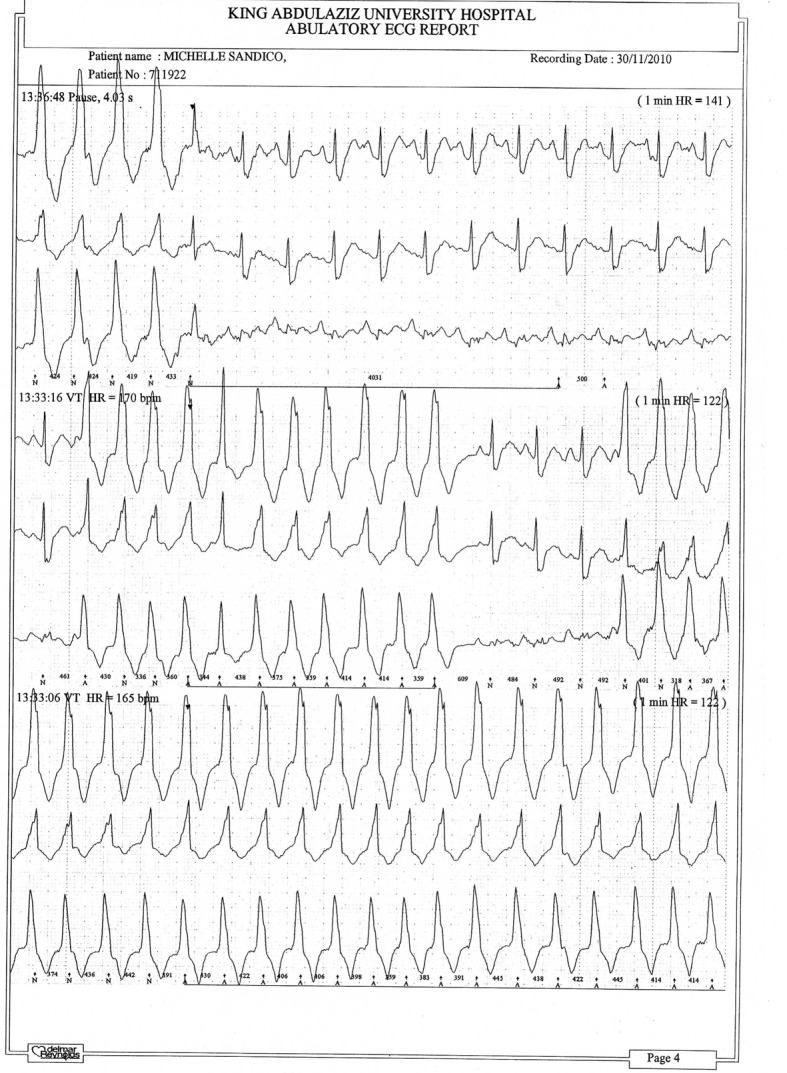
Part of the Holter monitor tracing of one patient before colon clear.

**Figure 2:**
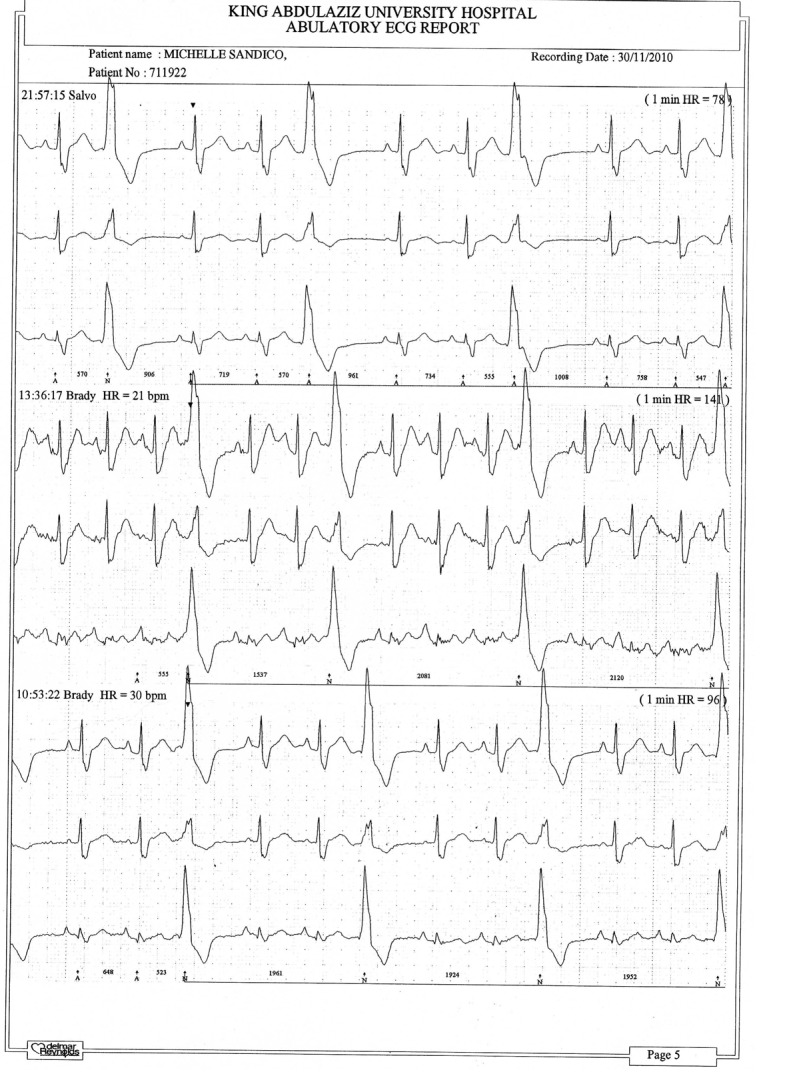
Another part of the Holter tracing of the same patient before colon clear.

**Figure 3:**
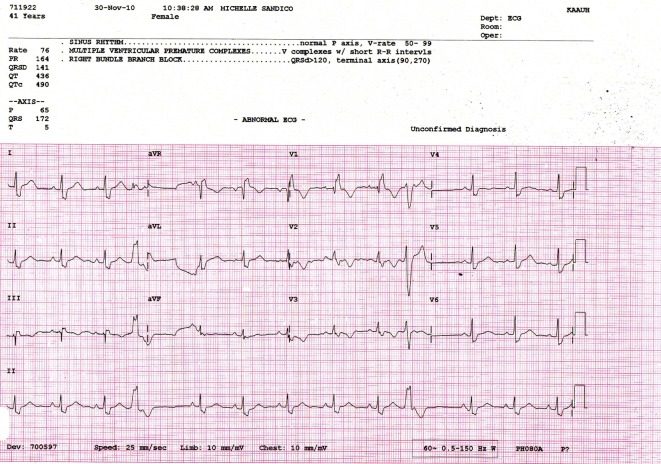
ECG tracing of the same patient before colon clear.

**Figure 4:**
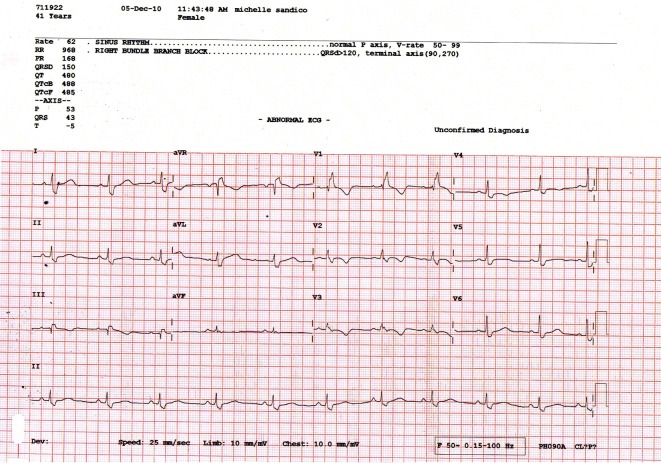
ECG tracing of the same patient after colon clear.

Figures 1 and 2 show parts of the Holter monitor tracing of one of the patients of the study before undergoing colon clear. Figure 3 shows the ECG tracing of the same patient before colon clear, while Figure 4 shows her ECG tracing after colon clear.

## Discussion

Migration of *H. pylori* to the colon is a fact that has been reported in literature [2-4]. It was suggested that the antibiotic violence could have forced the stomach bacterium to migrate to the colon rather than eradicating it from the stomach [2, 4]. This suggestion is supported by the finding that pseudo-membranous toxic colitis and toxic megacolon have developed after eradication of *H. pylori* from the stomach by antibiotic therapy [14, 15]. *H. pylori* in the colon will continue producing ammonia for a reason or no reason leading to accumulation of profuse toxic amounts of ammonia, unopposed or buffered by any acidity. Ammonia is known to be toxic and colon spastic [16]; a colonic re-absorptive error could establish leading to excessive fluid and salt retention in the body with subsequent burden on blood pressure and heart rate. Accumulation of ammonia in the colon could lead to adverse toxic effects on the myocardium in susceptible people; toxic myocarditis and cardiomyopathy could be integral sequels of these toxic effects [11, 12].

The results of this study confirm the concept of improvement of cardiomyopathy after colon clear due to elimination of a potential source of toxins from the colon as the wonderful senna purge is potent and is known to effectively kill and/or expel the migrated colonic *H. pylori* strains [4, 17]. This concept is further supported by the results achieved by the patient who followed the camel milk/honey therapeutic remedy; camel milk is a known potent colon clear measure while acetate which is directly lethal to *H. pylori* is existing among the end products of glucose utilization by the body [17-19].

In this study, the effect of the senna leaves extract on the growth of *H. pylori* was studied; addition of three times dilution of the senna leaves purge preparation to solid culture of *H. pylori* was found directly lethal to it.

The records of the seven female patients who followed medical treatment and AICD insertion in one of them failed to provide information about any history of *H. pylori* for those patients although it was expected as existence of abnormal *H. pylori* strains is common particularly in developing countries [2, 4, 17], and because of their failure to respond to adequate medications, most probably due to persistence of an underlying etiologic pathology.

Toxic myocarditis rather than viral myocarditis could have been the pathological etiology behind many cases diagnosed as idiopathic cardiomyopathy during the last three decades. The value of this study is gained from the true promising opportunity that many cases of cardiomyopathy could be avoided and that many cases of the newly discovered condition could be cured or at least progress of the disease could be stopped.

A general impression has developed that those patients developing cardiomyopathy due to the influence of colonic *H. pylori* strains are disadvantaged susceptible individuals; they should watch their colonic condition and employ colon care and colon clear whenever they develop frank dyspeptic symptoms. It was also observed that females are more predisposed than males to these adverse toxic effects produced by the abnormal-behavior colonic *H. pylori* strains; therefore, further comparative studies concerning this regard including both male and female patients are recommended.

### Conclusion

The senna leaves purge should be considered as a recognized potent colon clear measure. Toxic myocarditis rather than viral could be the pathologic etiology behind many cases of idiopathic cardiomyopathy; hence it could be prevented, the newly discovered conditions could be cured or at least progress of the disease could be stopped. Eradication of *H. pylori* from the colon via colon clear could lead to improvement of cardiomyopathy due to elimination of colonic *H. pylori* strains below its pathologic level. Colon clear could be a simple and safe measure to improve changes in cardiac rhythm, heart rate and myocardial function developing in association with *H. pylori* due to inflammatory, toxic or immune reasons.
